# Milk-borne bacterial health hazards in milk produced for commercial purpose in Tigray, northern Ethiopia

**DOI:** 10.1186/s12889-020-09016-6

**Published:** 2020-06-09

**Authors:** Gebretsadik Berhe, Araya Gebreyesus Wasihun, Enquebaher Kassaye, Kibrom Gebreselasie

**Affiliations:** 1 College of Health Sciences, Mekelle University, Mekelle, Ethiopia; 2 College of Veterinary Medicine, Mekelle University, Mekelle, Ethiopia

**Keywords:** Bacteria, Commercial, Health-hazards, Milk-borne, Tigray

## Abstract

**Background:**

Milk being a suitable medium for bacterial growth, it can serve as a source of bacterial contamination. Pathogenic bacteria in milk pose a serious health threat to humans and constitute about 90% of all dairy-related diseases. However, there are few studies that examined the health hazards of raw milk consumption in Ethiopia. Therefore, the objective of this study was to assess the prevalence of bacterial contamination and associated factors in milk produced for commercial purpose in Tigray region, northern Ethiopia.

**Methods:**

This study used a cross-sectional study design, selected 315 persons (168 cafeterias, 96 dairy farms, and 51 milk vendors) for interview and collected the same number of bulk raw milk samples using systematic sampling procedure. Data were collected on socio-demographic, farm hygiene and milk handling practices by trained health professionals. Bacterial contamination was defined as total bacterial count (TBC) > 1 × 10^5^, staphylococcus count (SC) > 10^5^, or coliform count (CC) > 10^2^ CFU/ml by culture and the species of bacteria were determined by standard biochemical tests.

**Results:**

From the 315 milk samples tested, the prevalence of bacterial contamination was 52% (95% CI: 46.5–57.6). The mean counts of contaminated samples of TBC, SC, and CC were 8.94 ± 0.46 Standard Deviation (SD), 8.52 ± 0.6 SD, and 8.78 ± 0.49 SD log CFU/ml, respectively**.** The proportion of contamination was significantly lower in milk collected from dairy farms (32/96, 33.3, 95% CI: 24.5–43.2) compared to milk from vendors (33/51, 64.7, 95% CI: 51.4–66.2) and cafeterias (99/168, 58.9, 95% CI, 50.9–76.85). The milk samples were culture-positive for *Escherichia coli*, *Klebsiella pneumoniae*, *Staphylococcus aureus*, *K. oxytoca* and *Citrobacter freundii*.

**Conclusions:**

Over half of the sampled raw milk exhibited bacterial contamination with increasing trend from farmers to points of sale. Thus, milk vendors and cafeteria owners should apply good hygienic and sanitation practices during handling of milk; use appropriate, clean containers, and cold chain during milk transportation; and refrigeration of milk during storage.

## Background

Milk and dairy products are important source of vital nutrients for human beings [1, 2]. The unique composition and properties make milk an excellent medium for bacterial growth and source of bacterial infection [3]. Milk-borne pathogenic bacteria pose a serious threat to human health, and constitute about 90% of all dairy- related diseases [4]. *Staphylococcus aureus, Salmonella spp., Listeria monocytogenes*, *Escherichia coli* O157:H7 and *Campylobacter* are the main microbiological hazards associated with raw milk consumption [3]. Microbiological status of raw milk is affected by several factors including a health status of the animal, farm management practices, environmental hygiene and poor temperature control [5].

In some countries with low socio-economic status, income growth and urbanization has led to almost doubled consumption of milk and dairy products [1, 2]. In Ethiopia, as the dairy industry is developing towards a market-oriented system, majority of milk vendors and cafeterias collect unpasteurized milk from different dairy farms to sell it to consumers [6]. A survey conducted in central Ethiopia reported that 31.8% of farmers consumed raw milk [7]. In the milk market value chain, milk handled in unhygienic way can be easily contaminated by milk-borne bacterial pathogens and serve as a suitable vehicle for disease transmission, causing a significant public health threat to consumers [6–8]. A study documented that hygienic practices during milking, handling and storing of milk were substandard resulting in poor quality milk products and safety problems [9]. A study conducted in northern Ethiopia reported milk contamination rate varying from 45 to 75% [10]. `.

In Tigray region, where less than 1% of the milk produced for commercial purpose is pasteurized and in situation where there is no set standard and regulation to control the safety and quality of milk produced for commercial purpose, the risk of milk contamination is high. Despite this fact, there are few studies that examined the health hazards of raw milk consumption in the study area, other than those conducted in localized areas with a small sample size. This study is expected to fill the gap in information on the magnitude and type of hazards associated with raw milk consumption and outline measures that are needed to be taken by milk producers, vendors and regulators to ensure the safety and quality milk in the milk supply value chain.

Therefore, the objective of this study was to assess the prevalence of milk-borne bacterial hazards and associated factors in commercially marketed raw milk in the Tigray regional state, Ethiopia.

## Methods

### Study design and setting

This study used a cross-sectional study design to assess the magnitude of milk-borne bacterial pathogens and associated factors in the study area during February to May 2017.

### Study area

Tigray regional state is located in the northernmost part of Ethiopia. The region has a total population of 6.2 million, 85% of which live in the rural areas and about 83% of the populations were farmers [11]. This study was carried out in the three big cities of the regional state; Mekelle, Wukro and Adigrat. Mekelle is the capital city of the region and Adigrat is the zonal capital for the eastern zone of the Tigray region [11].

### Source and study population

The source populations were all dairy farmers, milk vendors, and cafeterias who sold milk in different forms in the three selected cities. The study populations were all dairy farmers, milk vendors, and cafeterias who sold milk in different forms in the selected lowest administrative units (*Kebelles)* in Mekelle, Wukro and Adigrat cities during the study period.

### Sample size determination and sampling procedure

Sample size was estimated using a single population proportion formula with the following assumption: Confidence level = 95%, prevalence = 0.75 [10], margin of error = 0.05. Thus, the total sample size was calculated as 288 study subjects and milk samples. We allocated the sample size using proportional to population size of the randomly selected ten *Kebelles* from Mekelle and four *Kebelles* from each of the two other study sites. In all the selected *Kebelles*, systematic random sampling was used to select dairy farmers, milk vendors or cafeterias.

### Data collection tools and methods

#### Questionnaire survey

The instrument used for this study was developed by modifying from other studies [7–10, 12, 13]. The investigators used a pre-tested structured questionnaire and administered it through face-to-face interviews with dairy farmers, milk vendors, and cafeteria owners after obtaining written informed consent from each study subject. Checklist was also used to assess the farm hygiene status. Data were collected on socio-demographic factors, milking, milk handling and storage practices. Data collectors were trained on the objective of the study, content, and method of administration of the tool. Data were collected by BSc level health professionals under close supervision.

#### Milk sample collection and laboratory examination

A total of 315 milk samples were collected from all enrolled dairy farmers (96), milk vendors (51), and cafeterias (168) by conducting house-to-house visits. About 30 mL of bulk milk samples were collected aseptically from the containers used for storage of milk, put into a sterile universal bottle and placed in a cool box packed with ice packs. Milk samples were transported from the field to the laboratory within four hours of collection and stored at 4 °C in a refrigerator until testing.

### Laboratory tests for bacterial isolation and identification

To determine the bacteriological quality of the raw cow milk, total bacterial count (TBC), staphylococcus spp. count (SC) and coliform count (CC) were performed. Plate Count agar (HiMedia, India), the Baired-Parker Agar Base (Oxoid, UK) enriched with Egg Yolk Tellurite Emulsion (Damstadt, Germany) and the Violet Red Bile agar (MiMedia, India) were used for each procedure, respectively. Peptone water was used as a diluent for serial ten-fold dilutions.

The enumeration of total viable bacteria, staphylococcus spp. and coliform bacteria were performed following standard procedures [14, 15]. To describe briefly, for each sample a tenfold serial dilution, up to 10^− 6^ dilutions were prepared using peptone water. From each dilution, 1 ml was put in the center of the respective agar plates using micropipette and spread evenly to the whole plate using sterile bent glass rod. For each serial dilution, duplicate agar plates were employed. The inoculated agar plates were incubated at 37 °C for 24–48 h. Agar plates with 30–300 colonies were counted using colony counter as per the recommendation [14, 15]. For identification of bacteria to species level, colony morphology and different primary and secondary biochemical testes were employed. The detailed laboratory procedures used for isolation and identification of the bacteria is described elsewhere [5, 6, 14–19].

### Data management and analysis

The investigators entered the data into Epidata software and exported it to SPSS version 20 for analysis. Bacterial counts data were transformed to logarithm of colony forming units per milliliter of sample (log CFU/ml) and the results were presented as mean ± standard error (SE), median with interquartile range (IQR) and percentage (%). Bacterial contamination level in cow milk was graded according to the Standard European Union (EU) microbiological limits (TBC ≤1 × 10^5^ CFU/mL, SC < 10^5^ CFU/mL and CC ≤10^2^ CFU/mL) [20]. Descriptive statistics were calculated using simple frequencies, percentages, and mean. Bivariate and multivariable logistic regressions were used to identify factors associated with milk-borne bacterial hazards by controlling the confounding effect of selected variables. Statistical significance was determined by *p* values, crude odds ratio (COR) and adjusted odds ratio (AOR) with their corresponding 95% confidence intervals.

### Ethical considerations

Ethical clearance was obtained from the Ethical Review Board of the College of Health Sciences, Mekelle University. All the study subjects in this study were adults above 20 years old. The investigators administered written informed consent to all study subjects after provision of information on the study. The autonomy of study participants was ensured and all collected data were kept confidential.

## Results

### Socio-demographic characteristics of the study population

Data were collected from a total of 315 study subjects (96 dairy farmers, 51 milk vendors, and 168 cafeterias) in the three selected study sites. The median age of study participants was 37 years (IQR = 31–43 years) and ranged from 20 to 75 years. The highest number of study participants was from Mekelle city and the lowest from Wukro city. Male participants accounted for 55.6% of the study subjects (Table 1).

**Table 1:** Socio-demographic characteristics of study participants in the study area in 2017

Variable	Categories	Frequency	Percent
**Residence**	Mekelle	182	57.8
	Wukro	25	7.9
	Adigrat	108	34.3
**Age**	20 < 37	177	56.2
	38–75	138	43.8
**Sex**	Males	175	55.6
	Females	140	44.4
**Level of education**	illiterate	24	7.6
	1-8th Grade	171	54.3
	9th grade and above	120	38.1
**Marital status**	Single	94	29.8
	Married	209	66.3
	Divorced	12	3.8
	Widowed	0	0
**Occupation**	Dairy farmers	81	28.7
	Cafeterias	155	49.2
	Milk vendors	47	14.9
	Others	32	10.2
**Type of Businesses**	Dairy Farmers	96	31.5
	Vendors and cafeterias	219	69.5

From the 96 dairy farmers who practiced cleaning of utensils, 44.4% (43/96) used cold water and soap for washing utensils while 13.5% (13/96) used only cold water. All of the interviewed dairy farmers reported that they washed hands before milking and 68.8% (66/96) used cold water and soap for washing their hands. The udders of lactating cows were washed with cold water by 46.2% of the famers. All dairy farmers reported that they filtered the milk after milking and before selling to consumers. To transport milk to their customers, 69.7% of the dairy farmers reported that they used narrow necked plastic vessels and 25.0% used wide neck plastic vessels. Transporting the milk to market was done mainly on foot (57.3%) and bicycles (16.7%). The source of drinking water for the farms was mainly tap water (68.8%) followed by wells (16.7%) (Table 2).

**Table 2:** Hygienic practices of dairy farmers in the study area in 2017

No.	Description of variable	Frequency	Percent
1	**Cleaning of utensils**		
	Cold water	13	13.5
	Soap and cold water	43	44.8
	Soap and hot water	39	40.6
	Only hot water	1	1.0
2	**Hand washing**		
	Cold water	18	18.8
	Soap and cold water	66	68.8
	Soap and hot water	10	10.4
	Only hot water	2	2.1
3	**Udder washing**		
	Cold water	46	47.9
	Soap and cold water	9	9.4
	Soap and hot water	24	25
	Only hot water	17	17.7
4	**Containers used for transportation of milk**		
	Wide necked aluminum vessel	3	3.03
	Narrow necked aluminum vessel	0	0
	Narrow necked plastic vessel	69	69.7
	Wide neck plastic vessel	24	25.0
5	**Means of milk transport**		
	Cars	11	11.5
	Bicycle	16	16.7
	Bajaj or Motorcycle	14	14.6
	On Foot	55	57.3
6	**Sources of water**		
	Tap water	66	68.8
	Wells	16	16.7
	Ponds and streams	14	14.6
7	**Milk storage container**		
	Plastic container	92	94.8
	Stainless steel container	2	2.1
	Aluminum container	2	2.1

As regards to milk deliveries, about half (53.2%) of milk vendors and cafeterias received milk in the morning and in the afternoon, 43.1% of them received in the early morning and 3.7% received in the afternoon. Nearly half (48.6%) of the milk delivery was from one dairy farm. Moreover, 36% of milk vendors and cafeteria stored milk in a refrigerator and 23% of them stored it at room temperature. It was also found that 50% of the respondents had encountered milk spoilage (Table 3).

**Table 3:** Milk handling practices of milk vendors and cafeterias in the study area in 2017

Description of variable (***n*** = 216)	Frequency	Percent
**Time of milk collection**		
Evening	8	3.7
Early morning	93	43.1
Both	115	53.2
**Sources of milk**		
Own farm	30	13.8
Other one farm	105	48.6
Other two farms	38	17.6
Other three or more farms	40	18.5
Milk vendors	3	1.4
**Type of milk sold**		
Raw milk	7	3.2
Boiled milk	69	31.9
Yogurt	11	5.1
Boiled milk and yoghurt	84	38.9
Raw milk, boiled milk and yogurt	45	20.8
**Milk storage**		
Refrigerator	78	36.1
Room temperature	49	22.7
Boiling and room temperature	13	6.0
Boiling and refrigeration	76	35.2
**Time to finish the milk**		
One day after collection	137	63.4
Two days after collection	76	35.2
More than 2 days after collection	3	1.4
**Incident of milk spoiling**		
Yes	108	50
No	108	50

### Prevalence of bacterial contamination of milk

**Fig. 1:**
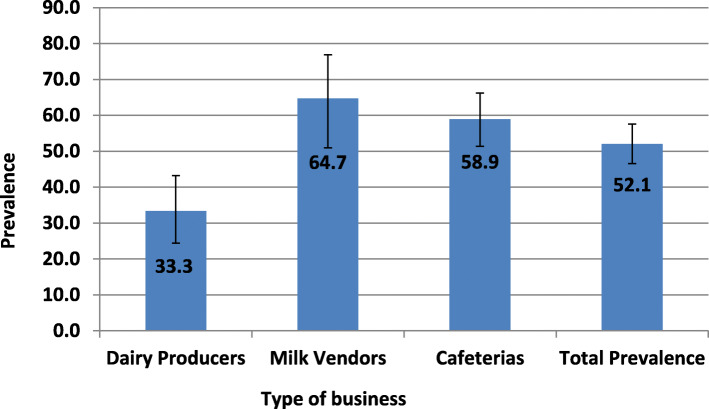
Prevalence of bacterial contamination by types of business in the study area in 2017

The prevalence of bacterial contamination of milk was 52% (95% CI: 46.5–57.6). The recorded proportion of contamination was highest in milk collected from vendors (33/51, 64.7%), followed by cafeterias (99/168, 58.9%) and dairy farms (32/96, 33.3%) (Fig. 1). However, there was no statistically significant difference in the proportion of contamination between milk collected from cafeterias (64.7, 95% CI: 50.9–76.85) and milk vendors (58.9, 95% CI: 51.4–66.2). Moreover, there was no significant variation in the proportion of milk contamination among milk collected from the three cities: Mekelle (56.6, 95% CI: 49.3–63.7), Wukro (48.0, 95% CI: 32.8–70.8) and Adigrat cities (45.4, 95% CI: 36.2–54.8).

Of the 315 cultured samples, 52.1% (164) of the total bacterial count and 40.6% (128) of Staphylococcus count exceeded the threshold limit of acceptable quality of milk and thus graded as contaminated or poor quality. Similarly, 47.9% (151) coliform counts had microbial count in excess of ≤10^2^ CFU/ml. The mean of contaminated total bacterial count, staphylococcus spp. count and coliform count was 8.94 ± 0.46 (Std. dev.), 8.52 ± 0.6 (Std. dev.) and 8.78 ± 0.49 (Std. dev.) log CFU/ml of milk, respectively**.**

**Fig. 2:**
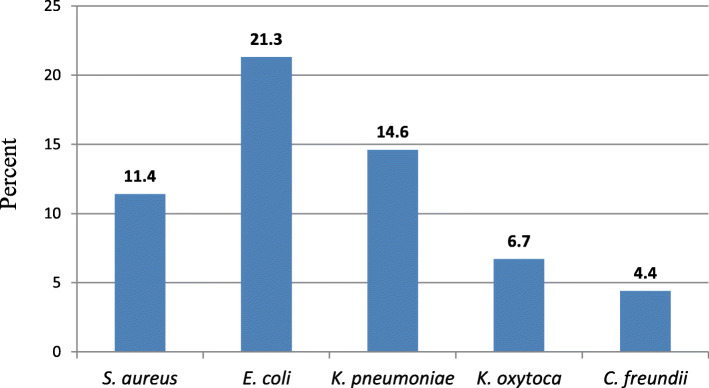
Type and proportion of isolated bacteria from milk samples collected from the study area in 2017

The milk samples were culture-positive for *E. coli*, *K. pneumoniae* and *S. aureus, K. oxytoca* and *C. freundii.* Among the identified bacteria, *E. coli* (21.3%), *K. pneumoniae* (14.6%) and *S. aureus* (11.4%) were isolated with highest proportion (Fig. 2).

### Factors associated with bacterial contamination of milk

In the bivariate and multivariable logistic regression analysis; variables including residence, age, sex, educational status, marital status and type of business were analyzed for possible association with contamination of milk. In the bivariate analysis, although the OR for 1-8th grade educational level was greater than one, the 95% confidence interval (0.58–3.39) was statistically insignificant. Similarly, sex and marital status were not significantly associated with increased prevalence of bacterial contamination. However, type of business was statistically associated with milk contamination both in the bivariate and multivariable logistic regression analysis. Milk collected from milk vendors and cafeteria owners were nearly three times more contaminated (AOR = 2.72, 95% CI = 1.60–4.61) as compared to milk sampled from dairy farmers (Table 4).

**Table 4:** Factors associated with increased prevalence of bacterial contamination of milk in the study area in 2017

Variable	Positive (%)	Negative (%)	COR	95% CI	AOR	95% CI
**City**						
Mekelle	103 (32.7)	79 (25.1)	1			
Adigrat	49 (15.6)	59 (18.7)	0.708	0.306–1.63		
Wukro	12 (3.8)	13 (4.1)	1.11	0.465–2.65		
**Age**						
20 < 37	95 (30.2)	82 (26.0)	1			
38–75	69 (21.9)	69 (21.9)	0.863	0.55–1.35		
**Sex**						
Male	84 (26.7)	91 (28.9)	1		1	
Female	80 (25.4)	60 (19.0)	1.44	0.924–2.26	1.01	0.618–1.67
**Educational status**						
Illiterate	10 (3.2)	14 (4.4)	1			
1-8th grade	94 (29.8)	77 (24.4)	1.4	0.577–3.39		
9th grade and above	60 (19.0)	60 (19.0)	0.819	0.513–1.31		
**Marital status**						
Single	61 (19.4)	33 (10.5)	1		1	
Married	98 (31.1)	111 (35.2)	0.386	0.144–1.31	0.542	0.152–1.94
Divorced	5 (1.6)	7 (2.2)	0.809	0.249–2.63	0.892	0.260–3.06
**Type of Business**						
Dairy farmers	33 (10.5)	63 (20.0)	1		1	
Vendors and Cafeterias	131 (41.6)	88 (27.9)	3.08	1.87–5.08	2.72	1.60–4.61

## Discussion

This study reported a higher prevalence of bacterial contamination of milk (52%). The prevalence of milk-borne bacterial contamination of milk is similar to a report from Adigrat city where milk contamination rate was 45% in farm settings, 60% in milk vendor shops and 75% in cafeterias [10]. In terms of *S. aureus* contamination level, it was lower than the prevalence of *S. aureus* (19.6%) reported in Central Oromia [21] and Tigray region (38.7%), Ethiopia [6]. Similarly, this study recorded lower contamination level comparing to a report from Côte d’Ivoire where 76.5% of the samples showed the presence of one or more of the three pathogens (*E. coli, S. aureus,* and *Enterococcus*) [22]. Moreover, higher rate of contamination of milk was reported from another study in India [23]**.** Possible reasons for this high level of milk contamination in the study area could be attributed to the use of un-pasteurized milk for commercial purposes, sub-optimal hygiene practices, inadequate cooling and lack of standard facilities for milking, storage, and transportation of milk. There is considerable evidence that microbial contamination in the milk market value chain can originate from a diseased cow, unhygienic milking practice, poor personal hygiene, unsanitary utensils and/or milking equipment, poor storage conditions, and lack pure water supply [24, 25]. A study reported that in developing countries like Ethiopia, there are inadequate hygienic practices throughout the dairy production system and standard milking protocols do not exist which is evidenced by the presence of many dairy farmers that do not disinfect teats prior to milking and inadequate washing of hands before milking [26, 27].

From the total cultured milk samples, 47.9 and 40.6% were isolates of coliform and *S. aureus*, respectively. Coliform counts are important indicators that are used to measure the level of microbial quality and hygiene in milk handling [28, 29]. Previous studies have also indicated that coliform and staphylococcus are the most frequent bacterial contaminants of milk and this was attributed to their abundant availability both in the animal body and in the environment [29, 30]. In this study, more than 31% of the dairy farms have been using rivers, ponds or wells as their source of water and this might have contributed to the high coliform contamination of milk in our study. Contamination of *S. aureus* can arise from various sources as the bacteria is commonly found on the various body parts of warm blooded animals and it can be isolated from faeces, soil and fresh water [25]. Moreover, *S. aureus* can be carried in *a*pproximately 20–50% healthy individuals in their nasal cavity [31].

The high bacterial contamination rate of milk in our study implies that milk can pose health risks to consumers. If milk is not handled properly and in hygienic condition, it will support the growth of pathogenic micro-organisms leading to transmission of zoonotic and foodborne diseases that can compromise the health of the population. Therefore, to prevent contamination of milk by pathogenic micro-organism, improving animal health, environmental hygiene, dairy farming practices, milk handling, transportation and storage practices are required [28, 32].

Among the socio-demographic factors evaluated for association with milk contamination, type of business was significantly associated with higher level of milk contamination. The proportion of milk contamination was significantly lower in milk collected from dairy producers (33.3, 95% CI: 24.5–43.2) compared to milk from vendors and cafeterias. This is consistent with the findings of Merhawit et al. [10] and Oliver et al. [33] where the bacteriological quality of milk deteriorated along the milk supply chain due to the proliferation of the microorganisms initially present in the milk or/and due to cross contamination [4]. As there was increasing trend of milk contamination from farmers to points of sale, milk vendors and cafeterias should use appropriate and clean containers, timely delivery of milk, cold chain during transportation, and refrigeration of milk during storage.

Throughout the developing world, over 80% of the milk consumed is unregulated, and in Ethiopia less than 1% of the milk consumed is pasteurized [34, 35]. It is reported that the contamination of milk is high in countries where there is traditional farming systems and market is unregulated. Increased contamination level of milk along the milk market chain indicates that food safety is becoming an important public health agenda particularly in milk produced for commercial purpose. When milk is produced for commercial purpose with inadequate hygienic practices, milk contaminated with pathogenic micro-organisms can end-up with mass distribution, mass outbreaks affecting more people and cause a greater economic impact. Therefore, focus needs to be placed on setting food safety standards particularly for milk produced for commercial purpose [4]. Generally, the implementation of good hygienic practice and sanitation, introduction of quality and safety standards as well as the use of an effective cold chain and pasteurization technologies are the key measures to improve the microbial quality and safety of the milk.

This study was conducted in a large milk-shed area using adequate sample size. As limitation, this study didn’t comprehensively examine all important bacterial contaminants of milk and factors responsible for milk contamination. Moreover, residual confounding and social desirability bias might have occurred during variables measurement.

## Conclusions

The following conclusions are made from this study:

The level of milk contamination in this study was high indicating the presence of significant health risks to the consumer. Milk collected from milk vendors and cafeterias was significantly associated with higher level of milk contamination compared to milk sampled from dairy farms.

Therefore, considering the study findings, the following recommendations are made.

Milk vendors and cafeteria owners should apply good hygienic and sanitation practices during handling of milk; use appropriate, clean containers, and cold chain during transportation; and refrigeration of milk during storage. Governmental bodies should set quality and safety standards for milk produced for commercial purpose to improve microbial quality and safety of milk. Further research is required to assess contamination of milk by other important pathogens particularly *Salmonella* spp., *Campylobacter* spp*., L. monocytogenes* spp*.*

## Data Availability

The datasets used and/or analyzed during the current study are available from the corresponding author on reasonable request.

## Abbreviations

AOR: Adjusted Odds Ratio
COR: Crude Odds ratio
CC: Coliform count
CFU: Colony forming unit
CI: Confidence interval
SC: Staphylococcus count
TBC: Total bacterial count
